# Acute Stroke in Tubercular Meningitis: A Case Report

**DOI:** 10.31729/jnma.4646

**Published:** 2019-10-31

**Authors:** Suzit Bhusal, Sujata Dahal, Neha Gautam, Prakash Banjade

**Affiliations:** 1 Kathmandu Medical College and Teaching Hospital, Sinamangal, Kathmandu, Nepal; 2 Manipal College of Medical Sciences, Pokhara, Nepal

**Keywords:** *meningeal tuberculosis*, *stroke; tuberculoma*, *vasculitis*

## Abstract

Most of the strokes in tubercular meningitis are multiple, bilateral, and located in the basal ganglia, especially the ‘tubercular zone’, which comprises of the caudate, anterior thalamus, anterior limb, and genu of the internal capsule. These are attributed to the involvement of medial striate, thalamotuberal, and thalamostriate arteries, which are embedded in exudates and likely to be stretched by coexistent hydrocephalus. Corticosteroids with antitubercular therapy were thought to reduce mortality and morbidity but their role in lowering strokes has not been proven. The mechanism of stroke in our case was vasculitis. Here, we are reporting a case of 22-years female patient with tubercular meningitis. She had complications of ischemic infarct and severe communicating hydrocephalus with a seizure disorder.

## INTRODUCTION

Tuberculous meningitis (TBM) is one of the most devastating presentations of tuberculosis (TB), which constitutes about 10% of all TB cases and is responsible for about 40% of the deaths due to TB in developing countries.^1^ The primary complications of TBM include cerebral stroke, hydrocephalus, and tuberculoma formation.^2^ Ischemic stroke can result from multiple etiologies. It can also be a complication of tuberculous meningoencephalitis and determines its outcome. Stroke secondary to tuberculous meningoencephalitis occurs in 30% cases in the basal ganglia region, unusually in the thalamus. The mechanism of stroke, in this case, was vasculitis. Cerebral infarction in TBM is usually related to necrotizing arteritis of the vessels of the Circle of Willis involved in the basal meningitis.^3^ We report an unusual case of a 22-years-old female with a diagnosis of tubercular meningitis. She had complications of ischemic infarct and severe communicating hydrocephalus with a seizure disorder.

## CASE REPORT

A 22-years-old woman came to our hospital with chief complaints of slurring of speech, hemiparesis, disorientation, confusion, and restless movement of the right half of the body. The restless movement of the right half of the body was followed by weakness of the left half of the body, which was insidious in onset, progressive in nature. According to the patient party, she was unable to perform normal daily activities with normal conversation five days ago. The weakness of the left half of the body first appeared in the left lower limb, followed by worsening of that of the left upper limb. The patient developed slurring of speech and global aphasia later. There was a history of fever for 20 days, which was intermittent; the maximum temperature recorded was 103 degrees F associated with chills, rigor and excessive sweating. The patient also complained of multiple episodes of vomiting. History of clenching of teeth, up rolling of eyes, and abnormal body movements was also present.

**Figure 1:**
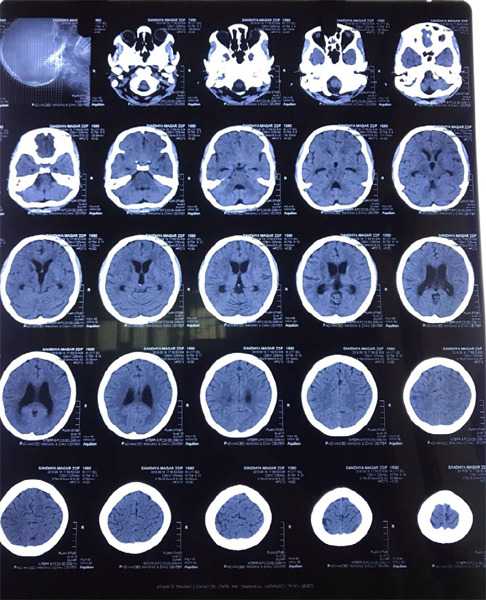
Axial plain CT scan of head showing infarct and communicating hydrocephalus.

The chest examination was normal. Cardiac rhythm and rate were normal with no murmurs. The abdomen was flat, soft, and non-tender with normal liver and spleen. Muscle strength of the right upper and lower limb was normal, but in the left upper and lower limb power was 0/5. Thalamic contracture of the right hand was also seen. All deep tendon reflexes were normal and plantar response was bilateral extensor. Signs of meningeal irritation were present. A computed tomography (CT) of the brain revealed infarct in the anterior limb of the right internal capsule and left the lentiform nucleus, bilateral lateral third ventricles including the 4^th^ ventricle, were dilated. That signified communicating hydrocephalus and infarct on the genu of the corpus callosum on the left side (Figure 1).

Cerebrospinal fluid (CSF) from lumbar puncture revealed specific gravity of 1.015, white cell count (WBC) of 3 cells/cumm/mm^3^, all lymphocytes, a protein level of 398.7mg/dl, a glucose level of 17mg/dl a serum glucose level of 130mg/dl and LDH of 467U/I. CSF fluid adenosine deaminase was 273mg/l. CSF was negative for bacterial culture, and KOH stain came negative. Her fasting blood glucose level was 226mg/dl; a known case of diabetes. The initial chest film showed no definite active lung lesion. Serology tests for Human Immunodeficiency Virus, Hepatitis B and C virus were negative. Moreover, liver and renal function tests were normal.

She developed hyponatremia 130meq/l and hypokalemia 3.4meq/l during the course of the disease which was corrected by IV fluid and KCI. Her liver function tests were normal. Based on clinical grounds and preliminary cerebrospinal fluid (CSF) laboratory results, antitubercular therapy was initiated. She was also started on dexamethasone and a combination of meropenem, vancomycin, acyclovir, and aspirin.

## DISCUSSION

Our patient illustrates that tubercular meningitis can have vascular complications. CNS tuberculosis (TB) includes three clinical categories: tuberculous meningitis (TBM), intracranial tuberculoma, and spinal tuberculous arachnoiditis.^4^ Thalamic contracture are induced by posterior thalamic lesions whereas they were on anterior nuclei in our patient. There is a poor correlation between neurological findings and focal lesions on imaging studies.^5^ Corticosteroids with antitubercular therapy were thought to reduce mortality and morbidity, but their role in reducing strokes has not been proven.^6^ Cerebral vasculitis is a rare disorder that causes inflammation of cerebral blood vessel walls. It has many different etiologies. Cerebral vasculitis due to tuberculous (TCV) is a catastrophic complication of TBM that accounts for secondary cerebral vasculitis.^7^

The cardinal features of TBM include fever, headache, malaise, confusion, meningeal signs and focal neurological deficits.^8^ Cerebral infarctions in TBM are found to occur primarily in the “TB zone,” which comprises of the heads of the caudate nuclei, the anteromedial thalami, the anterior limbs of the internal capsule, and the genua of the internal capsules.^9^ Common characteristics of tuberculosis patients include: having diabetes and coronary heart disease, living in the less urbanized areas, and having lower monthly income. The link between tuberculosis and coronary heart disease may be facilitated by diabetes, or chronic infections may take part in the pathophysiology of atherosclerosis and cardiovascular disease.^10^

In a case of a 15-year-old male with bilateral thalamic infarct complicating tuberculous meningitis, reported by Aasfara J et al.^11^ had a similar rise in CSF protein and WBCs. However, as in our case, bacilli culture in CSF was negative. The diagnosis was made based on findings of other investigations.
